# First evidence of microplastic inhalation among free-ranging small cetaceans

**DOI:** 10.1371/journal.pone.0309377

**Published:** 2024-10-16

**Authors:** Miranda K. Dziobak, Andreas Fahlman, Randall S. Wells, Ryan Takeshita, Cynthia Smith, Austin Gray, John Weinstein, Leslie B. Hart

**Affiliations:** 1 Department of Health and Human Performance, School of Health Sciences, College of Charleston, Charleston, SC, United States of America; 2 Department of Environmental Health Sciences, Arnold School of Public Health, University of South Carolina, Columbia, SC, United States of America; 3 Fundacion Oceanografic, Valencia, Spain; 4 Global Diving Research, Sanlucar de Barrameda, Spain; 5 IFM, Linkoping University, Linkoping, Sweden; 6 Chicago Zoological Society’s Sarasota Dolphin Research Program, ℅ Mote Marine Laboratory, Sarasota, FL, United States of America; 7 National Marine Mammal Foundation, San Diego, CA, United States of America; 8 Department of Biological Sciences, Virginia Tech, Blacksburg, VA, United States of America; 9 Department of Biology, The Citadel, Charleston, SC, United States of America; Animal Health Centre, CANADA

## Abstract

Plastic is a ubiquitous environmental contaminant, resulting in widespread exposure across terrestrial and marine spaces. In the environment, plastics can degrade into microparticles where exposure has been documented in a variety of fauna at all trophic levels. Human epidemiological studies have found relationships between inhaled microplastics and oxidative stress and inflammation. Previous studies of bottlenose dolphins (*Tursiops truncatus*) have reported prevalent exposure to plasticizing chemicals (e.g., phthalates) as well as particle loads in gastrointestinal tracts, but exposure from inhalation has not yet been studied. The objective of this study was to determine if inhalation is a viable route of microplastic exposure for free-ranging dolphins. Exhalation samples were opportunistically collected from dolphins residing in Sarasota Bay, Florida (n = 5) and Barataria Bay, Louisiana (n = 6) during catch-and-release health assessments to screen for microplastic particles. All dolphin samples contained at least one suspected microplastic particle, and polymer composition was determined for 100% of a subset (n = 17) of samples. Additional studies are warranted to better understand the extent of inhaled microplastics, as well as to explore impacts, given potential risks to lung function and health.

## Introduction

Microplastics (i.e., plastics < 5mm diameter) are ubiquitous pollutants; thousands of research studies are available on the topic [1–3] and document their presence in terrestrial [4–7], marine [8, 9], freshwater [10], and sea ice environments [11–13]. Previous studies have also tracked their abundance on every continent [14–19] and modeled how they can be transported through the air [20–22]. Because of this ubiquity, humans and wildlife are exposed through multiple mechanisms [23], but primarily via ingestion [24–27] and inhalation [28, 29]. Through ingestion and inhalation, microplastic deposition in the gut [30], stool [31], and lungs [32] are expected; however, these particles have also been detected in human heart [33] and placental tissue [34], as translocation and bloodstream transport can occur for very small microplastics and nanoplastics [35, 36]. Microplastics may also bioaccumulate in organisms; bioaccumulation potential was investigated in various trophic levels in a lake ecosystem in China, and organisms at the highest trophic levels were found to have the highest degree of microplastics [37]. These findings were consistent with other studies reporting microplastic bioaccumulation within trophic levels and in predator-prey relationships [38–40]. Conversely, no evidence of microplastic bioaccumulation within trophic levels has also been observed (e.g., [41], highlighting gaps in current knowledge of microplastic transport and fate. Further research is needed to better understand the role bioaccumulation and trophic transfer play in microplastic exposure, especially in consumers relying on fish and seafood as their main food source. In human epidemiological studies and experimental rodent studies, myriad adverse health impacts have been associated with microplastic exposure including oxidative stress and cytotoxicity [42–45], as well as inflammatory and immune responses related to altered gut microbiota diversity [46]. Additionally, environmental microplastics are often composed of thousands of synthetic chemicals (e.g., phthalates, bisphenol A (BPA), per- and polyfluoroalkyl substances (PFAS), brominated and organophosphate flame retardants [47]) and offer a high surface-to-volume ratio for the adsorption of other environmental contaminants (e.g., organic pollutants [48]; metals [49, 50]; harmful algal bloom toxins [49, 51, 52]; invasive species [53–55]; potentially pathogenic microbes [56, 57]. As a result, microplastics may be a vehicle of exposure to chemicals that have been associated with adverse influences on reproduction [58, 59], development [60], metabolism [61], and cardiovascular health [62, 63].

Human inhalation risk assessments have demonstrated higher particle abundance for indoor environments, a predominance of fragments and fibers, multi-polymeric composition, and particles smaller than 100 μm [64–69]. These risk assessment studies typically involve active and passive sampling equipment that collects air and dust, while human exposure has been directly quantified via sputum screening [70, 71], samples collected by nasal [71] or bronchoalveolar lavage [72], and investigations of lung fluid and tissue collected by surgical methods [28, 72]. Findings from these studies have demonstrated the propensity for both upper respiratory exposure [71] and a deeper infiltration within the respiratory tract, leading to particle accumulation in lung tissue [28, 72]. Although numerous factors, including occupation [73, 74], geographic location [75], and demography [25, 76], can impact microplastic inhalation, humans likely inhale hundreds of microplastic particles per day [25].

Despite abundant evidence of outdoor airborne microplastics [77, 78] and atmospheric fallout [22, 79–81], studies of microplastic inhalation in wildlife are nearly absent. This is especially surprising given widespread evidence of microplastic ingestion in marine wildlife (e.g., multiple fish species [82–84], shorebirds [85–87], sea turtles [88, 89], sharks [90–92], and marine mammals [26, 93–97] and recent studies suggesting that oceans may be sources of atmospheric microplastics [98, 99].

Bottlenose dolphins (*Tursiops truncatus*) inhabiting Sarasota Bay, FL, USA, have been the focus of health and population abundance studies since 1970 [100]. We recently presented evidence of prevalent exposure to ingested microplastics [94] and phthalate plasticizers [101–103]. Therefore, our objective was to determine if samples from exhaled air collected during routine catch-and-release health assessments [104, 105] can be used to identify and characterize microplastic inhalation exposure for free-ranging bottlenose dolphins.

## Materials and methods

### Sample collection

**Fig 1:**
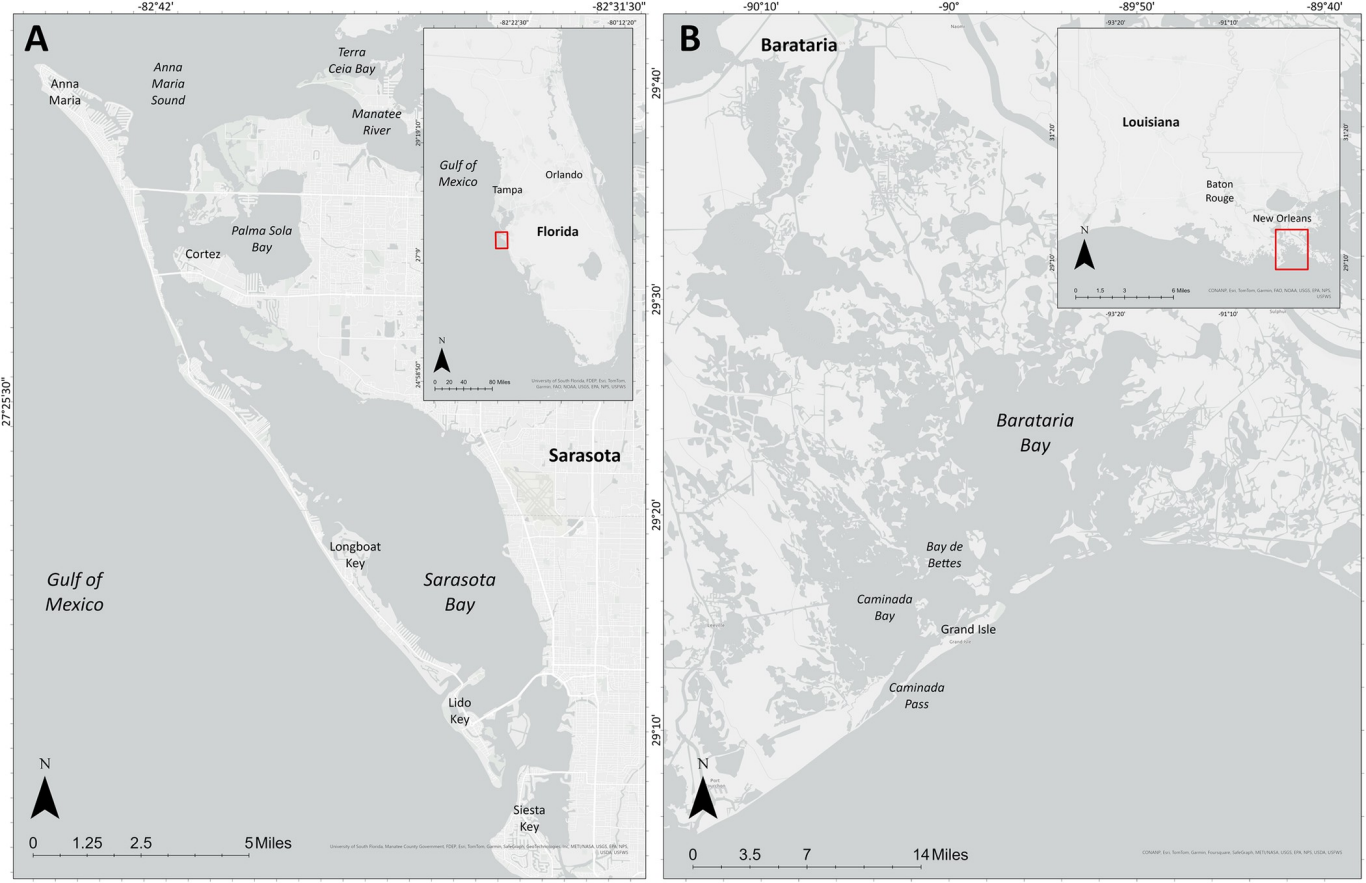
Bottlenose dolphin study sites: (A) Sarasota Bay, Florida and (B) Barataria Bay, Louisiana. Map created using Esri ArcGIS Pro basemap, Esri, TomTom, Garmin, FAO, NOAA, USGS, © OpenStreetMap contributors, and the GIS User Community [106].

Samples from exhaled air were collected from bottlenose dolphins during catch-and-release health assessments conducted in Sarasota Bay, FL, USA, (SB) and Barataria Bay, LA, USA (BB; Fig 1) in May and June 2023, respectively. During these health assessments, individual dolphins were encircled by a seine net and temporarily restrained to collect biological, physiological, and morphological data/samples indicative of their overall health. An experienced veterinary team attended to the dolphins during the examinations and sampling, to ensure their safety and welfare. In SB, exhalation samples were collected by holding a pre-cleaned (deionized (DI) water rinse) petri dish approximately 15 cm above the blowhole. To account for ambient particle contamination, a pre-cleaned petri dish was also held open next to the dolphin during sampling for use as a ‘field blank’. The field blanks were processed in the same way as the samples from exhaled air to account for any potential indoor ambient air contamination that may have occurred during laboratory procedures. Prior to sample collection, the blowhole and surrounding skin was gently dried with cotton gauze to prevent seawater contamination of the exhalation samples. Sample and field blank petri dishes were refrigerated until sample analysis.

For dolphins assessed in BB, samples from exhaled air were collected by rinsing the Fleish flow-cell that was housed inside a pneumotachometer used to evaluate lung function [107]. The flow-cell was rinsed with DI water after it was held over the blowhole to measure respiratory function. The DI water rinse was collected in a glass jar and refrigerated until sample analysis. To account for potential device contamination, the pneumotachometer was rinsed with DI water prior to the sampling of each dolphin; this DI rinse was used as the ‘field blank’.

Bottlenose health assessments were conducted under Scientific Research Permit #26622 (Sarasota Bay) and # 24359 (Barataria Bay), issued by the National Oceanic and Atmospheric Administration’s (NOAA) National Marine Fisheries Service (NMFS). Research studies were reviewed and approved by Mote Marine Laboratory (Sarasota Bay) NMFS Atlantic (Barataria Bay) Institutional Animal Care and Use Committees (IACUC). The Navy’s Bureau of Medicine and Surgery reviewed and approved the NMFS IACUC protocol for the Barataria Bay health assessments and issued a separate Navy protocol (NRD1265). The lung function studies were approved by the Animal Care Committee of the Oceanografic (Approval number: OCE-2v-19).

### Sample analysis

For microplastic processing, pneumotachometer rinses were filtered onto GF/A 1.6 μm glass fiber filters in a fume hood. Petri dish samples were rinsed three times with DI water onto GF/A 1.6 μm glass fiber filters in a fume hood. Samples were left to dry in covered glass petri dishes. Microplastic characterization followed criteria determined by [94]. Briefly, visual identification occurred for particles at least 35 μm in size using a dissection microscope (Leica EZ4, magnification 8-35x). Plastic composition was suspected for particles that melted or were marked when approached with a hot needle (250°C, ‘suspected microplastic particles’; [108, 109]. Suspected microplastic particles were categorized following previously defined criteria [94] based on physical attributes of the individual particle, including shape (e.g., fiber, film, fragment, foam), and color (e.g., transparent, blue, black; [110]. Visual characteristics were also used to further identify suspected plastic particles. For example, fibers were indicated by a smooth, uniform surface that was longer than it was wide [111], while fragments were indicated by smooth or angular edges that appeared to be broken from a larger piece of debris [111]. Fragments could be further classified as a film or a foam if they were flexible and able to be folded (film) or were distorted when handled but returned to the original shape (foam; [111]). Microplastic dimensions were measured using the Leica LAS EZ (version 3.4.0, Switzerland) imaging software system.

A subset of suspected microplastic particles (n = 17) from dolphin samples was analyzed for polymer type by Raman microspectroscopy (Xplora Plus with LabSpec 6 software version 6.5, Horiba Scientific). The subset was chosen based on the predominant types of particles found and placed on double-sided tape for analysis. The spectra were obtained using a 50X or 100X objective lens with a 785 nm or 532 nm laser at 0.1% to 100% power, confocal slit width of 100 μm, hole diameter of 300 μm with gratings of 600 or 1200 grooves/mm and 4s acquisition time. Raman spectral matches were processed using LabSpec6 and matched to OpenSpecy software to determine if particles were plastic or of anthropogenic origin [112]. All of the particles analyzed were matched with spectra from OpenSpecy. For our analysis, OpenSpecy had a Pearson r coefficient cut-off of 0.60. Our average Pearson r coefficient was 0.82 among all our samples.

### QA/QC and data analysis

Given the potential for airborne plastic contamination, precautions were taken to ensure sample integrity. A 100% cotton lab coat and clean nitrile gloves were worn during all laboratory analyses. All tools and glassware used in the laboratory were thoroughly rinsed with DI water. Field blanks were collected for each sampled dolphin and processed the same way as the samples to account for any contamination resulting from ambient indoor air. Three positive controls with commercially purchased microplastic particles (e.g., polyethylene and polyester) were used to determine recovery efficiency, and mean recovery percentages exceeded 80% for all control particles. As this was an exploratory study, descriptive statistics were used to summarize particle counts, suspected microplastic shapes, and polymer composition within and between sampling sites. Microplastic particle counts were compared between demographic characteristics (e.g., female vs. male; adult vs. subadult) with a Mann-Whitney U test. Sexual maturity (as determined by several factors including age, calving history, pregnancy diagnosis via ultrasonography, testis size from ultrasound, and sex hormone concentration; [113, 114]), was used to differentiate adults vs. subadults.

## Results

Exhalation samples, and matched field blanks, were collected from five SB and six BB dolphins in 2023. Microplastic particles isolated from field blanks included fibers, films, and fragments of multiple pigments, but most of the particles observed in SB field blanks were not similar to particles detected in dolphin samples (Table 1). Among BB samples, however, there were more similarities between exhalation samples and field blanks (Table 1). Suspected MPs identified in exhalation samples were corrected for field contamination by removing particles of the same shape and color from total particle counts (Table 2).

**Table 1:** Suspected microplastics identified in Sarasota Bay (n = 5) and Barataria Bay (n = 6) bottlenose dolphin exhalation samples.

SB Dolphin ID	Most Common Particle(s)	Fibers										Fragments		Films			
		R	Y	G	B	Pu	Pi	Bk	T	Yd	Br	G	Pu	Y	B	T	Br
F295	Fiber	1	1		2			2	2								
*Blank*					1					1							
F277	Fiber	1		1		7*		3				8*					
*Blank*					2												
F292	Fiber			1	1	8*	1					8*		1			1
*Blank*		1				10*						9*					
F297	Fiber			3	2			2			4						
*Blank*					3												
F326	Fiber					1		2	3								
*Blank*								2									
**BB Dolphin ID**	**Most Common Particle(s)**	**Fibers**										**Fragments**		**Films**			
		**R**	**Y**	**G**	**B**	**Pu**	**Pi**	**Bk**	**T**	**Yd**	**Br**	**G**	**Pu**	**Y**	**B**	**T**	**Br**
YR4	Fiber		1		5			5	2		1					1	
*Blank*					1		1	1	2								
YR2	Fiber		1		7	1		11	8		1						
*Blank*		2			5			8	8								
Y1F	Film	3			2			10	7						8		
*Blank*			1		2			9	6								
Y71	Fiber	2	1		6			1	2								
*Blank*			1		2		1		2								
Y73	Fiber	1	1		4	1		4	2				4				
*Blank*		1			1			1	1				2				
Y9F	Fiber, Film		1		2			8								1	
*Blank*					1			1									

*Used or recovered as positive controls during laboratory processing.

Colors: R-red; Y-yellow; G-green; B-blue; Pu-purple; Pi-pink; Bk-black; T-transparent; Yd-yellowed; Br-brown

Highlighted cells indicate particles that were not observed in QA/QC blanks or used as positive controls.

**Table 2:** Field blank-corrected particles observed in Sarasota Bay (n = 5) and Barataria Bay (n = 6) bottlenose dolphin exhalation samples.

**SB Dolphin ID**	**Most Common Particle(s)**	**Fibers**										**Fragments**		**Films**				**Total**
		**R**	**Y**	**G**	**B**	**Pu**	**Pi**	**Bk**	**T**	**Yd**	**Br**	**G**	**Pu**	**Y**	**B**	**T**	**Br**	
F295	Fiber	1	1					2	2									6
F277	Fiber	1		1				3										5
F292	Fiber			1	1		1							1			1	5
F297	Fiber			3				2			4							9
F326	Fiber					1			3									4
**Total**		**27**										**0**		**2**				**29**
**BB Dolphin ID**	**Most Common Particle(s)**	**Fibers**										**Fragments**		**Films**				
		**R**	**Y**	**G**	**B**	**Pu**	**Pi**	**Bk**	**T**	**Yd**	**Br**	**G**	**Pu**	**Y**	**B**	**T**	**Br**	
YR4	Fiber, Film		1								1					1		3
YR2	Fiber		1			1					1							3
Y1F	Film	3													8			11
Y71	Fiber	2						1										3
Y73	Fiber		1			1												2
Y9F	Fiber, Film		1													1		2
**Total**		**14**										**0**		**10**				**24**

Colors: R-red; Y-yellow; G-green; B-blue; Pu-purple; Pi-pink; Bk-black; T-transparent; Yd-yellowed; Br-brown

**Fig 2:**
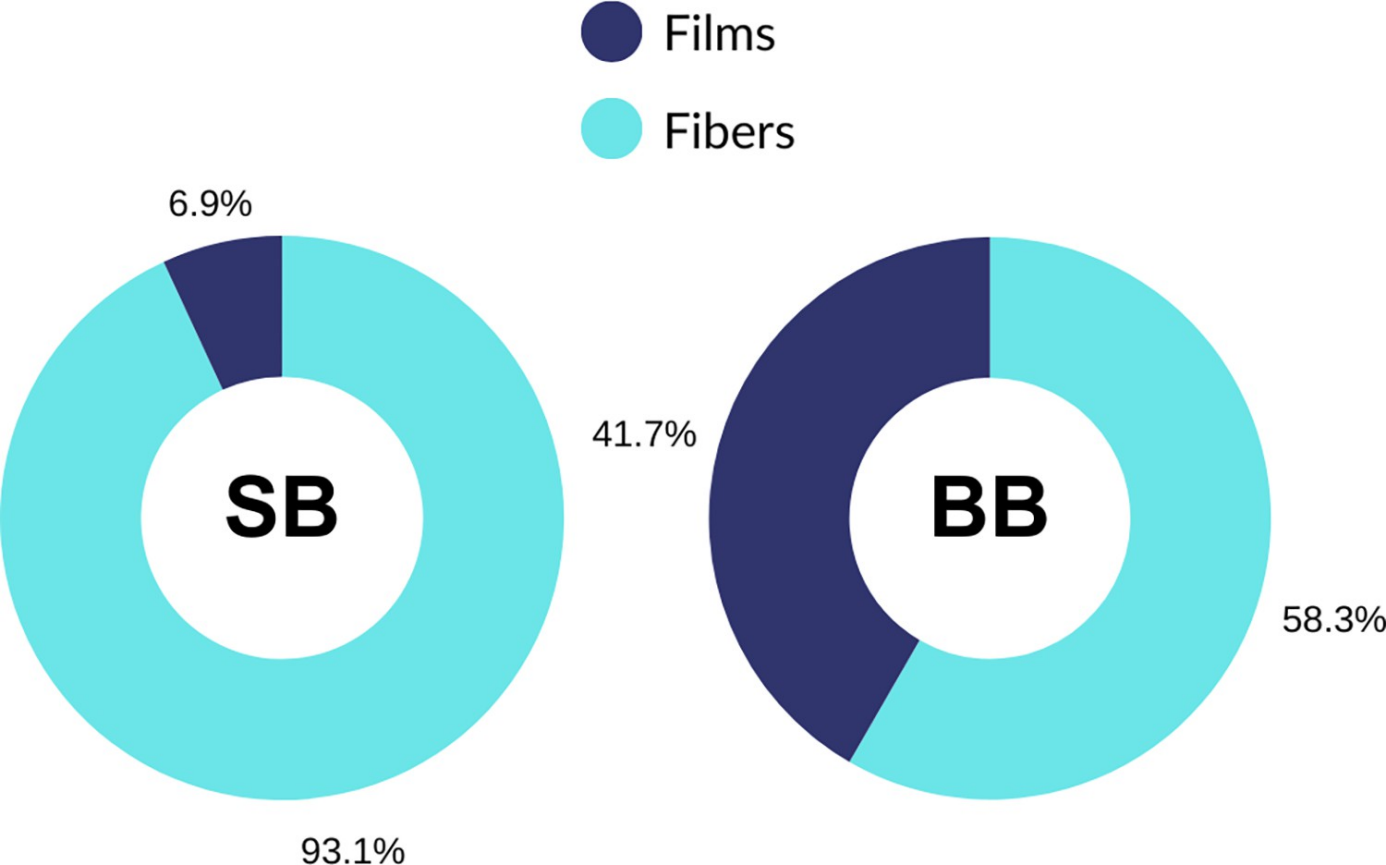
Proportion of microplastic particle shapes detected in bottlenose dolphins from Sarasota Bay, Florida, and Barataria Bay, Louisiana.

Following blank-correction, suspected microplastics were present in 100% of samples collected for this study (n = 11), and all particles were less than 500 μm in size. Fiber lengths ranged between 0.237 μm—1.7041 mm, and widths ranged between 0.0108 μm—0.0839 μm. Film widths ranged between 0.0437μm—0.172μm. Overall, there were 54 unique particles across all exhalation samples, and a nearly even distribution between sites (SB: 29; BB: 25; Table 2). The proportion of fibers and films detected between sampling sites was significantly different (Yates χ2 = 7.19, df = 1, p = 0.007); fibers were more abundant in SB samples (93%), while BB particles were composed of films (44%) and fibers (56%; Fig 2). In addition to differences in particle shape between sites, pigment predominance also varied. SB fibers were mostly green, black, transparent, and brown; BB fibers were mostly red and yellow. There are only two films observed in SB samples (yellow and brown), and most BB films were blue (Table 2).

Particle counts were compared between dolphins sampled at each location (Table 3). There were no differences determined between demographic characteristics (i.e., sex or age class) of the dolphins; however, given our small sample size, this finding may not be representative of either population.

**Table 3:** Demographic characteristics of bottlenose dolphins sampled from Sarasota Bay (SB), Florida and Barataria Bay (BB) Louisiana.

Dolphin	Total Particles	Site	Sex	Age Class
F295	6	SB	Female	Subadult
F277	5	SB	Female	Adult
F292	5	SB	Male	Adult
F297	9	SB	Female	Subadult
F326	4	SB	Male	Subadult
YR4	3	BB	Male	Adult
YR2	3	BB	Male	Adult
Y1F	11	BB	Female	Adult
Y71	3	BB	Female	Adult
Y73	2	BB	Female	Adult
Y9F	2	BB	Female	Adult
*p* *	*-*	*0*.*08*	*0*.*65*	*0*.*19*

*calculated using Mann-Whitney U test

**Fig 3:**
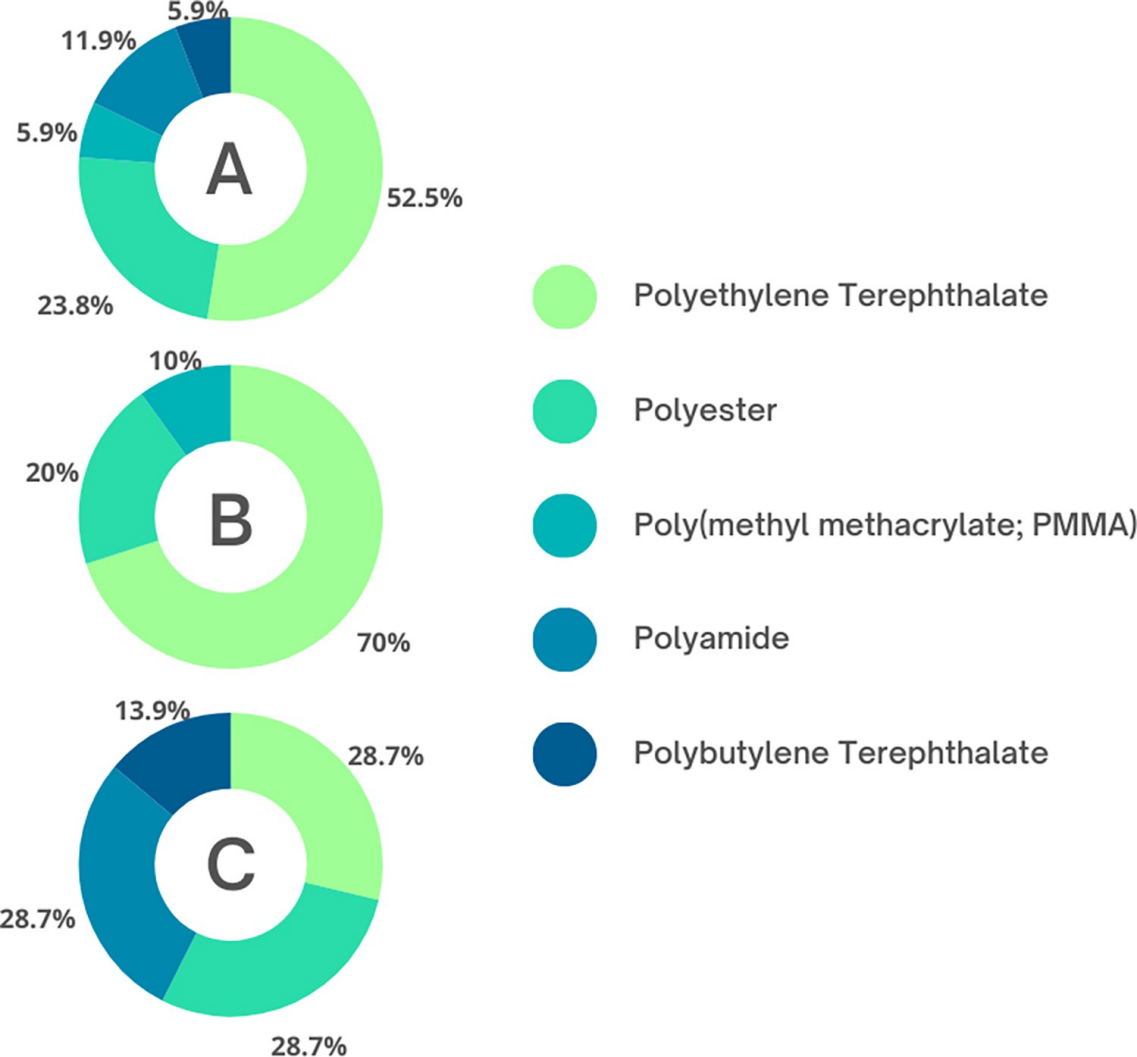
Polymer detection in microplastic particles from bottlenose dolphin exhalation samples in (A) all sampled individuals, (B) dolphins from Sarasota Bay, Florida, and (C) dolphins from Barataria Bay, Louisiana.

A subset of suspected microplastic particles (n = 17; 31.5% of total unique particles) from dolphin samples was analyzed for polymer type. Raman analysis demonstrated that 100% of the particles analyzed were confirmed to be of plastic origin (Table 4). Of the 17 particles, 58% were fibers, and 42% fragments. The most dominant particle identified was polyethylene terephthalate (PET), making up 53% of the microplastic particles analyzed (Table 4). Polyester (PE) was the second most dominant particle type, making up 24% of the particles (Table 4, Fig 3). Other polymers identified included polyamide, polybutylene terephthalate, and poly(methyl methacrylate; PMMA), representing 12%, 6%, and 6% of particles analyzed, respectively (Table 4, Fig 3). Given the variability in polymer type identified and the small sample size, we were underpowered to conduct further stratified comparison studies of polymers detected within and between sampling sites.

**Table 4:** Particle and polymer type identified in exhalation samples from individual dolphins residing in Sarasota Bay, Florida and Barataria Bay, Louisiana. Data obtained from OpenSpecy [112].

SB Dolphin ID	Particle type	Polymer type	Pearson r coefficient
F295	Fiber	Polyethylene Terephthalate	0.93
F292	Fragment	Polyester	0.77
	Fiber	Polyethylene Terephthalate	0.92
F326	Fiber	Polyethylene Terephthalate	0.9
F297	Fiber	Polyethylene Terephthalate	0.88
	Fiber	Polyethylene Terephthalate	0.91
F277	Fiber	Polyethylene Terephthalate	0.88
	Fragment	Poly(methyl methacrylate)	0.59
	Fiber	Polyethylene Terephthalate	0.94
	Fiber	Polyester	0.91
**BB Dolphin ID**	**Particle type**	**Polymer type**	**Pearson r coefficient **
YR4	Fragment	Polyethylene Terephthalate	0.72
Y73	Fiber	Polyethylene Terephthalate	0.88
Y1F	Fragment	Polyester	0.86
	Fragment	Polyamide	0.74
	Fragment	Polyamide	0.67
	Fragment	Polybutylene terephthalate	0.67
	Fiber	Polyester	0.79

## Discussion

### Microplastic inhalation in free-ranging bottlenose dolphins

To our knowledge, this is the first study to identify and characterize microplastic inhalation exposure in a free-ranging marine mammal. Suspected microplastics were identified in all exhalation samples collected from dolphins residing in Sarasota Bay, FL (n = 5), and Barataria Bay, LA (n = 6). A subset (n = 17) of suspected particles was analyzed with Raman spectroscopy, and plastic composition was confirmed for all samples. These findings suggest inhalation as a mechanism of microplastic exposure for marine mammals.

Airborne microplastic particle distribution and deposition has been reported in both urban and rural areas [115]; therefore, it was expected that microplastics are present in both of our field sites. Environmental contamination by fibrous microplastics is common in urban areas, potentially due to sewage wastewater emissions [116–118]. Evidence provided by [99] shows the potential for sea spray to release microplastics into the atmosphere, so any wastewater discharged into SB could contribute to detection in those dolphins. Although Sarasota operates an urban reclaimed water transmission system [119], temporarily failing, or overwhelmed systems (such as the Bee Ridge Wastewater Reclamation Facility, which has released more than 800 million gallons of reuse water into the bay since 2013; [120]) could result in wastewater discharges into the bay. Further, positive correlations have been observed between particle abundance and human activity [115, 116, 121]. While microplastics are common in urban areas with high levels of human activity, they have also been detected in rural environments with limited human activity [122]. Microplastic particles (microfibers in particular) originating in urban environments have been shown to transport through the atmosphere to reach extremely remote regions of the globe [123], so it seems possible that the proximity of BB to larger cities such as New Orleans (population ~370,000; [124]) could therefore increase vulnerability to microplastic contamination.

Conversely, polymeric composition of inhaled microplastics was different between sampling sites. Of fibrous particles detected in exhalation samples, the majority (80%) were comprised of PET. This is consistent with previous studies observing PET abundance in marine atmospheric microplastic particles (e.g., northern Atlantic Ocean [125], South China Sea [126–128], Baltic Sea [129], the west Pacific Ocean [128] and the Indian Ocean [127]). While PET was detected among samples from both sites, it was much more prevalent in SB particles (70% vs. 29%; Fig 1). PET is most commonly used to produce textiles and fabric [130]; therefore, it would be expected that urban areas susceptible to wastewater discharge (especially from clothes washing) would experience greater PET contamination compared to rural areas. Previous studies conducted in China have linked PET detection with urbanization and increased population density [131, 132] suggesting links between human activity and prevalent contamination. PET has also been detected in atmospheric fallout [79] and deposition [133] in rural sites, thus providing a plausible exposure route for BB dolphins. In addition to PET, polyethylene (PE) was also detected in microplastic particles from SB and BB exhalation samples (20% and 29%, respectively). PE is also commonly used in clothing production and is therefore found in wastewater [117, 118], as well as atmospheric deposition samples [134]. Findings from this study were consistent with human studies of inhaled microplastics [28, 70, 72] in which PET and PE were consistently detected more frequently than other polymer types. Although preliminary, these findings suggest widespread environmental contamination and potential vulnerability to adverse health outcomes related to microplastic exposure.

### Health implications

Microplastic toxicity is a function of polymer type [135], chemicals added during production (e.g., phthalates [136]), and adsorbed materials (e.g., organic pollutants [137]). Gaps in knowledge regarding inhalation as an exposure mechanism further complicates understanding health risks. Although available data are limited, inhaled microplastics are suspected to influence lung health. For example, oxidative stress and inflammation induced by microplastic exposure has been shown to result in pulmonary fibrosis in laboratory rodent studies [138–140]. In humans, pulmonary fibrosis is a progressive lung disease with poor prognosis and high mortality risk [141]. Dolphins rely on lung compression and collapse during diving, the capacity of which could be reduced by fibrosis [142]. Additionally, the depth at which lungs compress and collapse determines gas exchange, which would be limited by fibrosis as well [142]. Therefore, inhaled microplastic particles could pose a serious threat to pulmonary health. Microplastic inhalation may also offer an exposure route for other organs in the body. For example, microplastics have previously demonstrated translocation across lung epithelial cells to secondary organs (e.g., liver, kidney, brain [143]). From there, microplastics can induce oxidative stress and inflammation [144–146], leading to downstream tissue damage and increased risk of neoplasia [44]. This inflammation mechanism was demonstrated in laboratory rodent studies in which inflamed ovaries and reduced oocyte quality were attributed to microplastic exposure [147]. As this is the first study to document microplastic exposure via inhalation in a free-ranging cetacean species, further research is needed to understand if this exposure route contributes to or exacerbates the risk of adverse health impacts from microplastics alone, or as part of multiple stressor scenarios.

### Strengths and limitations

Plastic composition of suspected MPs was confirmed using Raman spectroscopy, a robust analytical technique that is commonly used in investigations of microplastic polymer types (e.g., [148–150]. Raman spectroscopy involves irradiating a sample with a light source (such as a laser) to provide information on the molecular vibrations [151, 152]. The spectral outputs from this process can be compared to a reference database to facilitate polymer identification. Outputs generated with Raman spectroscopy can be regarded similarly to a human fingerprint, where spectra are unique to each chemical structure [152]. Contamination by ambient microplastic particles during sample collection, processing, and analysis is a significant concern when conducting microplastic assessments. However, we employed rigorous QA/QC protocols to limit any potential contamination including use of 100% cotton lab coats, clean nitrile gloves, and DI rinsed glassware during all laboratory procedures. We also collected a blank with every exhalation sample to account for potential contamination in either the field or in the lab. Further, for data analysis, we employed a conservative approach to blank-correction samples, in which particle shapes and colors observed in blanks were removed from total counts of dolphin samples.

This study relied on opportunistic sampling of dolphins from both study sites; therefore, findings may not be representative of either dolphin population. Our small sample size further limits extrapolations to the larger populations. We also were only able to examine exhaled microplastics. There is the potential for differential deposition of various particles along the respiratory tract which could impact the particle types we detected. Although this has not been studied for microplastics specifically, other particle studies conducted in humans have determined deposition to be size dependent. For example, a previous study reported that 10μm particles will deposit in the mouth, while 0.25μm and 0.1μm particles will deposit in the lungs and alveolar regions respectively [153]. This has not yet been explored in dolphin respiratory tracts and requires further research. Despite efforts to account for ambient contamination, we did observe higher contamination among samples collected via pneumotachometer than petri dish. This could be a result of storage; the pneumotachometer is stored in a case where it is still potentially exposed to ambient particles, while the petri dishes remain sealed until use. To mitigate, a conservative blank correction protocol was employed to ensure that any particles detected in exhalation samples were unique to that sample and not likely to be a result of contamination. Future sampling via pneumotachometer should ensure adequate rinsing of the device prior to use to limit contamination. To standardize across future potential field projects, we recommend sample collection be conducted via petri dish.

## Conclusion

Inhaled microplastic particles were detected in all sampled dolphins from Sarasota Bay, Florida (n = 5) and Barataria Bay, Louisiana (n = 6). Findings from this study indicate inhalation as a relevant microplastic exposure route for bottlenose dolphins. Given significant gaps in knowledge regarding the health effects posed by inhaled particles, implications for these findings are unknown; however, laboratory rodent and human epidemiological studies suggest lung damage as a possible outcome of this exposure route. The potential for particle translocation into other tissues presents further opportunities for health risks throughout the individual. For areas like BB, inhaled microplastics are particularly concerning as wildlife in this area experienced myriad health impacts due to the Deepwater Horizon oil spill. Poor pulmonary health has been reported in Barataria dolphins related to the spill [154], so inhaled microplastic particles could exacerbate existing lung disease. Dolphin respiratory function can be measured using spirometry techniques [155] and could be used during health assessments to help evaluate how microplastics exposure may contribute to adverse health effects and respiratory disease in individual dolphins over time. Further research to understand health implications following inhaled microplastic exposure is warranted, especially among vulnerable populations experiencing adverse pulmonary impacts. A systematic assessment of particle exhalation in Sarasota Bay dolphins is planned to examine life history influences on exposure and associations with health impacts in the well-studied Sarasota Bay dolphin community.
